# Pembrolizumab microgravity crystallization experimentation

**DOI:** 10.1038/s41526-019-0090-3

**Published:** 2019-12-02

**Authors:** Paul Reichert, Winifred Prosise, Thierry O. Fischmann, Giovanna Scapin, Chakravarthy Narasimhan, April Spinale, Ray Polniak, Xiaoyu Yang, Erika Walsh, Daya Patel, Wendy Benjamin, Johnathan Welch, Denarra Simmons, Corey Strickland

**Affiliations:** 1 Computational and Structural Chemistry, Merck & Co., Inc., Kenilworth, NJ USA; 2 Sterile Formulations Sciences, Merck & Co., Inc., Kenilworth, NJ USA; 3 International Space Station National Laboratory Integration, Melbourne, FL USA; 4 CSC Quality Assurance, Washington, DC USA; 5 Biologics and Vaccines Formulation-Process Characterization, Merck & Co., Inc., Kenilworth, NJ USA; 6 Biologics and Vaccines Formulation-Potency and Functional Characterization, Merck & Co., Inc., Kenilworth, NJ USA

**Keywords:** Biophysical chemistry, Biochemistry

## Abstract

Crystallization processes have been widely used in the pharmaceutical industry for the manufacture, storage, and delivery of small-molecule and small protein therapeutics. However, the identification of crystallization processes for biologics, particularly monoclonal antibodies, has been prohibitive due to the size and the flexibility of their overall structure. There remains a challenge and an opportunity to utilize the benefits of crystallization of biologics. The research laboratories of Merck Sharp & Dome Corp. (MSD) in collaboration with the International Space Station (ISS) National Laboratory performed crystallization experiments with pembrolizumab (Keytruda^®^) on the SpaceX-Commercial Resupply Services-10 mission to the ISS. By leveraging microgravity effects such as reduced sedimentation and minimal convection currents, conditions producing crystalline suspensions of homogeneous monomodal particle size distribution (39 μm) in high yield were identified. In contrast, the control ground experiments produced crystalline suspensions with a heterogeneous bimodal distribution of 13 and 102 μm particles. In addition, the flight crystalline suspensions were less viscous and sedimented more uniformly than the comparable ground-based crystalline suspensions. These results have been applied to the production of crystalline suspensions on earth, using rotational mixers to reduce sedimentation and temperature gradients to induce and control crystallization. Using these techniques, we have been able to produce uniform crystalline suspensions (1–5 μm) with acceptable viscosity (<12 cP), rheological, and syringeability properties suitable for the preparation of an injectable formulation. The results of these studies may help widen the drug delivery options to improve the safety, adherence, and quality of life for patients and caregivers.

## Introdution

Monoclonal antibody (mAb) therapeutics have made a major impact on treating oncological, cardiovascular, metabolic, and neurological diseases and disorders.^1^ Currently, there are over 70 therapeutic mAbs marketed in the United States and Europe. Given the increasing use of mAb therapeutics, it is becoming evident that there is a need for improvements in the manufacture, delivery, and storage of mAbs. From a manufacturing point of view, mAb drugs are complicated to make and usually purified by multiple chromatographic steps.^2^ The final formulations require refrigeration and have limited shelf lives, impacting the overall cost of the treatment. Small-molecule drugs and peptide therapeutics like insulin are often purified and formulated for oral or parenteral administration using crystallization processes. These processes have been shown to reduce production cost as well as improve the overall quality and shelf life of the final formulations. Insulin crystallization, for example, has been used for over 60 years in manufacturing and delivery.^3^ On the other hand, for larger proteins, crystallization has been used primarily for structure determination using X-ray crystallography,^4^ and the methodology has focused solely on the production of large, single, and highly ordered crystals.^5^ While there is ongoing research into the application of crystallization for bioseparations^6^ as well as technology for large-scale crystallization,^7,8^ examples of protein crystallization processes for large-scale manufacturing applications are very limited.

Prior to final formulation (drug product), the mAb drug substance (active pharmaceutical ingredient) must be stored and/or shipped to worldwide sites. Often the drug substance is stored as frozen diluted solutions or frozen lyophilized powders.^9^ For areas of the world where refrigeration is limited, a product that is stable and can be reformulated at room temperature would be highly desirable; stable crystalline drug substances could be the answer.

Today, typical mAbs are administered as intravenous (IV) infusions in hospital settings, which impacts the quality of life for patients and the caregivers. The overall process may require patients to take days off to travel far distances for hospital care, which may also expose them to infections, and impacts caregivers who are accompanying them for their scheduled infusions. The IV infusion process entails a saline wash step, the mAb formulation infusion step, and followed by a final saline wash step. The overall procedure takes multiple hours.^10^ Additionally, a large subset of patients requiring mAb therapy cannot physically support IV infusions due to their vascular condition. They require a surgically implanted, subcutaneous (SC) port. A septum containing catheter connects the port to a vein through which drugs can be injected. The scheduling and implantation of the port often delays mAb therapy and increases the risk of infection.^11^ For this reason, there is ongoing research into the development of highly concentrated mAb formulations for SC injection,^12^ administered at a local doctor’s office requiring less frequent dosing. A typical therapeutic dose can be in the 150–200 mg range. Most mAbs have limited solubility and the viscosity of the solution has been shown to increase dramatically above 100 mg/ml concentration.^13^ A preferred SC formulation would be a 150–200 mg dose in a 1 ml volume. One potential advantage of using crystalline formulations is that they are often less viscous than the comparably concentrated solution formulations. For example, crystalline suspensions of mAbs such as Infliximab at 150 mg/ml have an acceptable viscosity value of 26 cP vs. an unacceptable 275 cP at the comparable solution concentration.^14^ There is limited information on the side effects or pathology of SC delivery of mAbs. However, a histochemistry study in mice was performed with crystalline trastuzumab, where no observed inflammation at the injection site nor any abnormalities in tissue samples was observed as compared to control tissue within normal limits.^14^

For these reasons, the use of crystallization processes for purification, drug delivery, and storage of mAbs is being investigated using Pembrolizumab (Keytruda^®^). Keytruda^®^ is an anti-programmed cell death protein-1 (PD-1) therapy that works by increasing the ability of the body’s immune system to help detect and fight tumor cells. Keytruda^®^ is a humanized mAb that blocks the interaction between PD-1 and its ligands, PD-L1 and PD-L2, thereby activating T lymphocytes. Keytruda has been approved in the United States for several cancer indications, including non-small-cell lung cancer, melanoma, urothelial bladder cancer, head and neck squamous cell cancer, Hodgkin’s lymphoma, microsatellite instability cancer, and gastric cancer, and is under review in several additional countries.^15^

The three-dimensional structure of Pembrolizumab, the first reported structure of a full-length humanized IgG4 mAb, was determined by X-ray crystallography,^16,17^ from crystals grown under high salt conditions. These high salt crystallization conditions were judged to be unsuitable for formulation and/or delivery applications, which require Generally Recognized As Safe excipients^18^ and stability in an iso-osmotic formulation. Using a discovery crystallization strategy, a polyethylene glycol (PEG) condition, which was deemed suitable for drug delivery applications, was identified, but required further optimization and development to become an efficient batch crystallization process. Optimization of the initial discovery condition (20 mM HEPES, pH 6.8, 15% PEG 3350) led to a final isotonic formulation consisting of 50 mM HEPES, pH 7–8, 8–10% PEG 3350.

To identify some of the key variables for crystal growth, microgravity experiments were utilized to investigate the effects of sedimentation rate and temperature gradients. Some information regarding the growth of large single crystals for crystallography under microgravity was already available from the numerous microgravity protein crystallization experiments that were run in the space shuttle era and onboard the International Space Station National Laboratory (ISS-NL).^19^ The research laboratories of Merck Sharp & Dome Corp. (MSD) have performed microgravity experiments on 12 previous Space Shuttle flights. These experiments were designed to explore microgravity effects for multiple pharmaceutical applications primarily using a small protein therapeutic, α-interferon (Intron A^®^). Space-grown crystalline suspensions of α-interferon from STS-70, for example, produced crystalline suspensions of higher quality and uniformity compared to earth-grown crystals, and were used in multiple primate pharmacological studies.^19^ Based on this earlier work, the goal was to gather more knowledge about the production of mAb crystals and crystalline suspensions with improved properties for pharmaceutical applications.

This is the first report of a microgravity crystallization experiment of a full-length mAb, which produced a homogeneous crystalline suspension with improved viscosity and rheological properties. In contrast, a bimodal crystalline suspension was derived from the control, ground-based experiment. By manipulating variables such as sedimentation rate and temperature gradients, we were able to reproduce in ground-based experiments similarly homogeneous crystalline suspensions.

Our SpaceX-Commercial Resupply Service-10 experiment was developed using the Handheld-Protein Crystallization Facility (HH-PCF) hardware, and it was the first large-scale batch crystallization experiment planned since the Space Shuttle era insulin and α-interferon experiments.^20^ The MSD PCG (ISS-NL PCG-5) payload was launched on 19 February 2017 on SpaceX-10 and returned on 19 March 2017 with the SpaceX-10 Dragon capsule.

**Fig. 1:**
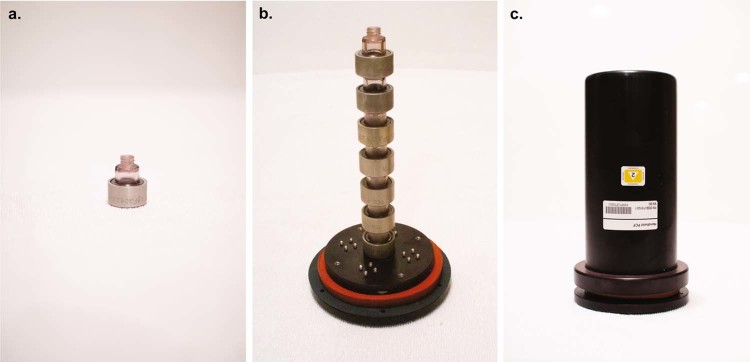
HH-PCF hardware: **a** 1 ml polysulfone bottle with aluminum cap. **b** Base plate with one tower of 7 × 1 ml polysulfone bottles with aluminum caps and orange gasket for sealing. **c** Outer aluminum cover, which covers the base plate.

The HH-PCF hardware was developed by the Center for Biophysical Sciences and Engineering at the University of Alabama at Birmingham and was designed to facilitate the production of crystals and crystalline suspensions.^21^ The HH-PCF hardware consists of an outer case containing five towers each with seven bottles. Hence, each HH-PCF system contains 35 individual protein crystal crystallization experiments, each within its own bottle. Experiment solutions were prepared ahead of time and stored at a specified temperature (between −95 and 37 °C). Figure 1 shows a single bottle, bottle tower, and the outer assembly.

**Fig. 2:**
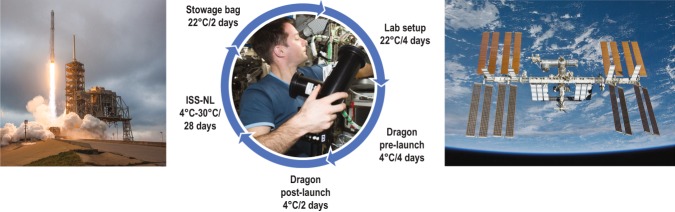
Experiment overview: Illustration of the timing and sequence of the overall experiment process from lab setup to recovery of the stowage bag. Center photo insert; Astronaut Thomas Pesquet (European Space Agency) removing the HH-PCF assemblies for return in stowage bag. Falcon-9 launch image on right is permissible to use within the public domain, courtesy of SpaceX. The center photo insert was obtained by written informed consent from Thomas Pesquet. The left image of the International Space Station is permissible within the public domain, courtesy of NASA.

Both the flight and ground experiments were setup in parallel in the Space Life Science lab prior to launch. The space experiment was turned over to Cold Stowage (a dedicated NASA ISS team responsible for providing a controlled temperature environment) and SpaceX for integration into the Dragon capsule. Upon Dragon docking at the ISS, the experiment was transferred to a Microgravity Experiment Research Locker Incubator (MERLIN) for activation using a controlled temperature ramp. The experiment remained in the MERLIN for 18 days prior to being placed back in a stowage bag onboard the returning Dragon capsule. Figure 2 illustrates a schematic overview of the timing and processing of the experiment.

## Results

### Experimental setup

The MSD PCG payload focused on the microgravity crystallization of pembrolizumab using the HH-PCF hardware module. A ground control experiment was setup using the same pembrolizumab and crystallization reagents and was activated and characterized in parallel to the flight experiment. A batch crystallization method was devised using 50 mM HEPES, pH 7.7, 100 mM caffeine, 10.8% PEG 3350, and the pembrolizumab concentration was varied between 20 and 40 mg/ml; a temperature ramp (4–30 °C over 48 h) was used to induce crystallization. The size of these experiments (1 ml bottles) was chosen to produce enough crystalline suspension for characterization using standard methods to identify crystals and analyze their rheological behavior and bioactivity. For flight and ground experiments, visual inspection, crystallinity, particle size, viscosity, sedimentation rate, and bioactivity measurements were used to document and characterize the contents of each bottle.

### Visual inspection and crystallinity

**Fig. 3:**
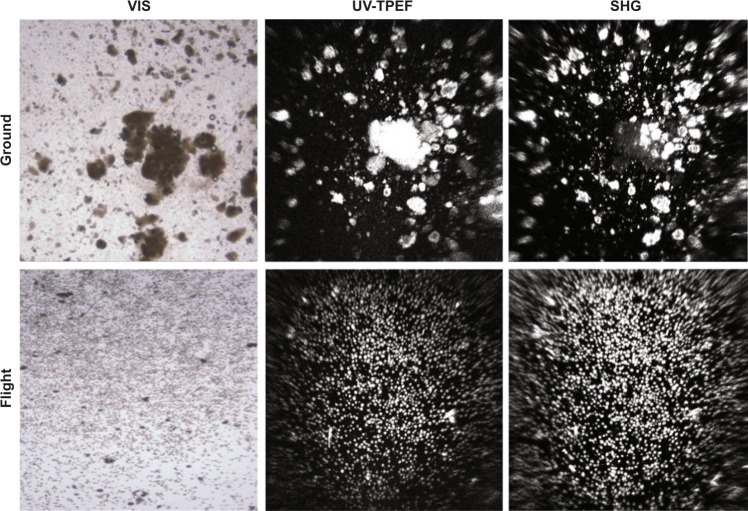
SONICC analyses: visible, UV two-photon excited fluorescence and second-harmonic generation images for the ground and flight experiments. Particles were confirmed to be proteinous based on positive UV. There are 22 trytophans in pembrolizumab and their crystals give a strong UV signal. The pembrolizumab crystals are chiral and therefore give a strong SHG signal. Although the shown images are at slightly different focal planes, all the observed visible particles were UV and SHG positive. The upper panel is a representative sample of bottle contents from a ground experiment by visible (×200), UV, and SHG imaging. The bottom panel: the visible, UV, and SHG image from a representative flight experiment.

Crystalline suspensions were observed in all bottles (flight and ground) and further documented by low-resolution photo imaging. Representative samples of each bottle were analyzed for crystallinity using second-order non-linear imaging of chiral crystals (SONICC) imaging technology.^22^ The observed particles were confirmed to be proteinaceous using ultraviolet two-photon excited fluorescence (UV-TPEF) imaging, and to be chiral crystals using second-harmonic generated (SHG) imaging. Both techniques generate a positive image of particles vs. a black background (Fig. 3).

### Particle size analysis

**Fig. 4:**
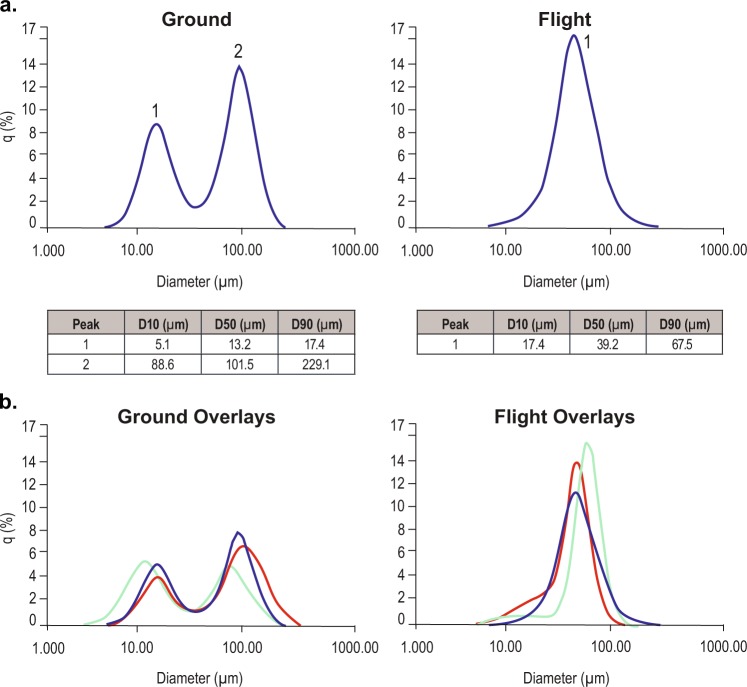
Comparison of ground (left column) and flight (right column) bottles by particle size analyses of same bottles (**a**). The data are presented graphically as *q*%, which is the density distribution at a size vs. the particle size diameter in micrometers. Below is a table of the distribution D10, D50, and D90. The DX is defined as the diameter where *X*% of the population lies below this value. The particle size analyses overlays (color coded) from three independent ground and flight experiments are shown in **b**.

To further characterize the possible differences between the flight and ground experiments, particle size analyses were run on the contents of each bottle using a laser diffraction particle size-distribution analyzer: Horiba LA-960.^23^ Laser diffraction analyses are based on the theory that a particle will scatter light at an angle determined by that particle’s size. Larger particles will scatter at small angles and smaller particles scatter at wide angles. A collection of particles will produce a pattern of scattered light defined by intensity and angle that can be transformed into a particle size-distribution result. The particle size density graph data is displayed graphically, *q*% is the probability area density distribution vs. the particle size diameter in micrometers. The contents of each bottle were thoroughly mixed and analyzed under constant stirring at room temperature in triplicate. There was a striking difference in the particle size and size distribution for the ground vs. flight bottles. Particle size analyses are shown Fig. 4a. The experiments were very consistent; ground bottles showed a heterogeneous bimodal distribution of sizes, which could be modeled using a bimodal model with maximum distribution around 13 and 102 μm, whereas the flight bottles showed a homogeneous distribution of particles that could be modeled as monomodal distribution centered around an average size of 39 μm. The reproducibility of the results from multiple bottles is shown in Fig. 4b.

### Dynamic viscosity measurement

Viscosity measurements^24^ were run in triplicates. Samples were prepared by combining and concentrating by centrifugation the crystalline suspensions of four bottles each from comparable flight and ground experiments and resuspending it in a stabilization solution (20 mM HEPES, pH 6.8, 10% PEG 3350). The final concentration was adjusted to 50 and 75 mg/ml and analyzed using a Rheosense m-VROC^TM^ viscometer. The average viscosity for the ground 50 mg/ml crystal suspension concentrate (CSC) was 5.48 ± 0.24 cP vs. the 3.67 ± 0.20 cP measured for the comparable flight experiment, a difference of 1.8 cP. For the ground 75 mg/ml CSC, the measured viscosity was 6.83 ± 0.10 cP vs. the 4.80 ± 0.01 cP measured for the comparable flight experiment, again a difference of 2 cP. The viscosity for the vehicle control was measured at 3.14 ± 0.01 cP. Thus, both 50 and 75 mg/ml flight crystalline suspensions have a 2 cP lower viscosity to the comparable ground experiment (Table 1).

**Table 1:** M-VROC viscosity measurements ground vs. flight at 50 and 75 mg/ml CSCs

Sample	50 mg/ml CSC viscosity (cP ± geometric SD)	75 mg/ml CSC viscosity (cP ± geometric SD
Ground	5.48 ± 0.23	6.83 ± 0.10
Flight	3.66 ± 0.20	4.80 ± 0.01
Δ Ground vs. flight	1.82 ± 0.03	2.03 ± 0.09
Vehicle (control)	3.14 ± 0.0.1	3.14 ± 0.01

*CSC* crystalline suspension concentrate

### Sedimentation time and dynamic light scattering measurements

Since it is desirable for an injectable product to have a composition of particles with consistent and predictable rheological properties, the sedimentation time and aggregation state of both flight and ground samples was assessed. The measured sedimentation time run in triplicate for the 50 mg/ml CSC flight sample was 57 ± 2 min, while the 50 mg/ml CSC ground sample did not fully sediment even after several hours. These results are consistent with the particle size analyses showing that the flight crystals with a homogeneous monomodal particle size (39 μm) sediment uniformly, whereas the ground crystals with a heterogeneous bimodal distribution in size (13 and 102 μm) sediment in a non-uniform, gradient-like manner over a longer period. Dynamic light scattering studies^25^ of CSC of both the flight and ground samples dissolved in saline phosphate buffer resulted in monodisperse solutions with an average 150,000 MW (Da) (the calculated MW for pembrolizumab is 146,252 Da) and a polydispersity index of 5.9% and 4.3%, respectively. Polydispersity indexes <15% are consistent with monodisperse protein solutions. These results demonstrate that both samples show similar dissolution properties compared to a control pembrolizumab solution with an average 150,200 MW (Da) and a polydispersity index of 5.9%. Thus, the crystallization process does not increase the propensity for aggregation by DLS analyses.

### Bioassay data

**Fig. 5:**
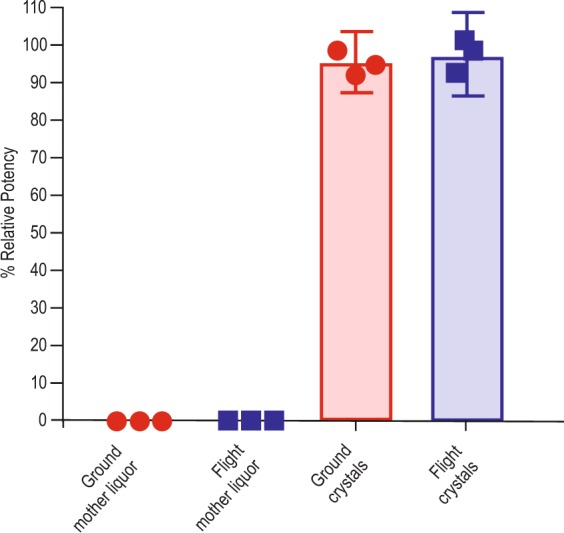
Competitive binding assay of flight and ground dissolved crystals and complimentary mother liquors. Dissolved crystals contain binding activity >94% relative to reference pembrolizumab (*N* = 3, 95% CI).

Representative samples from each flight and ground module were analyzed in a pembrolizumab enzyme-linked immunosorbent assay (ELISA) binding assay.^26^ The geometric mean of relative potency from multiple replicates (*N* = 3) of the same sample is reported with geometric standard deviation (%GSD) and 95% confidence interval. The potency of pembrolizumab samples in a competitive binding ELISA is shown in Fig. 5. These results demonstrated that the overall process (crystallization, dissolution, and subsequent handling) did not negatively affect the pembrolizumab competitive binding functionality in either the flight or ground experiments within the error of the pembrolizumab ELISA binding assay.

### Application to laboratory crystallization processes

At least 48 variables have been identified, which affect protein crystal growth.^27^ For the pembrolizumab crystallization condition investigated, sedimentation and temperature were identified as key variables in microgravity crystal growth. Experiments were devised to explore if these effects could improve crystal growth on earth. To minimize sedimentation a digital bottle roller with slow and horizonal rotation set at 24 r.p.m. (Labnet Hybridization Oven) was utilized. This method allowed the microcrystals, which contain 50–70% water, to remain buoyant during the process, thereby reducing sedimentation. This rotation speed was identified by visual observation of the experiment: at slower rotation rates, crystalline particles were observed to sediment during crystallization; at higher revolutions, the crystalline particles pelleted on the walls of the crystallization vessel. At the 1 ml scale, batch crystallization experiments carried out using a 4–22 °C temperature gradient over 24 h to induce crystallization and the digital bottle roller resulted in uniform crystalline suspensions with a geometric mean of 1.4 ± 1.7 μm; under identical but static (no rotation) conditions, the crystalline suspension had a more diverse particle size distribution with a geometric mean size of 4.7 ± 10.5 μm. The use of vertical rotation in the same Labnet hybridization oven resulted in less uniform particle size distributions using the 4–22 °C temperature gradient over 24 h.

To test the effect that the temperature gradients may have on the particle size distribution, we attempted to extend the temperature gradient over a period longer than 24 h, without using any rotation. However, using the 4–30 °C gradient over 24 h, there was no improvement on the particle size distribution observed, possibly due to agglomeration effects as a result of sedimentation. However, we observed that using an inverted temperature gradient, especially a 50 to 22 °C decrease over 24 h, resulted in more uniform crystalline suspensions with a geometric mean particle size of 1.3 ± 0.5 μm. These results suggest that crystallization of temperature-sensitive proteins could benefit in utilizing a temperature gradient-based strategy to improve crystal nucleation and growth behavior, which is not usually considered in protein crystallization studies. Results of these studies are summarized in Table 2.

**Table 2:** Applied sedimentation and temperature gradient effects on particle size distribution

Effect	Temperature gradient	Geometric mean particle size (μm ± geometric SD)
Rotation (24 rev/min)	4–22 °C/24 h	1.4 ± 1.7
No rotation	4–22 °C/24 h	4.7 ± 10.5
Temperature gradient	4–22 °C/48, 72 h	36 ± 2.9
No rotation		
Inverted temperature gradient	50–22 °C/24 h	1.3 ± 0.5
No rotation		

In summary, the flight experiments produced a monomodal population of crystalline particles as compared to the ground-based experiments, which produced a bimodal particle size distribution, reproducibly. Flight concentrated crystalline suspensions were less viscous than the comparable ground-based experiments in rheological studies. The concentrated flight samples sedimented more uniformly than the comparable ground-based experiments. Both flight and ground samples were shown to have comparable activity in the competitive binding assay.

## Discussion

The goal of this project was to understand which variables could affect mAb crystallization, using microgravity experiments as a research tool to identify better conditions for pembrolizumab crystallization. These microgravity experiments enabled the identification of sedimentation and temperature gradients as key variables to control crystal nucleation and growth for producing uniform crystalline pembrolizumab suspensions. These effects were tested and confirmed using standard laboratory ware. These results were consistent with earlier microgravity crystallization experiments with α-interferon where uniform particle size distributions were produced.^19^

The observed narrow particle size distribution in the flight experiment appears to be a consequence of a combination of minimal sedimentation and convection effects, resulting in a single nucleation and growth event with desirable viscosity and rheological properties.

There are several challenges of performing microgravity research, including adapting earth processes to flight-certified hardware, experimental timelines (setup and recovery), and real-time analysis. These factors can usually be easily addressed in earth experiments. However, designing and planning microgravity experiments forces more critical evaluation of factors not normally accounted for in earth experiments Nevertheless, microgravity research offers researchers an opportunity to generate materials distinct from classical earth-based experiments and provide unexpected results that can change the design and planning of earth-based experiments and processes.

Up to now, microgravity research has been severely limited by the available technology: for example, the only existing flight hardware for protein crystal growth is the one designed in the space shuttle era for the growth of relatively large single crystals for neutron and X-ray crystallographic structure determination studies rather than for crystalline suspensions. The hardware only allows for “black box” experiments, which require setup several days prior to activation, allow for analysis only upon recovery, and provide no opportunity to continuously monitor the experiments or to do iterative experiments during flight. In addition, there has been limited information on fluid dynamics and properties under microgravity conditions. To address this issue, a novel experiment was performed by NASA astronaut Kate Rubins during her 2016 mission to the ISS, demonstrating that liquid handling operations can be carried out in space using standard laboratory containers and procedures comparable to earth processes. The availability of specifically designed three-dimensional printed lab equipment (hardware) can also greatly enhance the rate of space research. Ideally, this could lead to the establishment of a dedicated laboratory area within the space station, where multiple experiments and analyses can be performed using standard scientific instrumentation. Most importantly, direct involvement of scientist-astronauts in experimental design, execution, and analyses provides the opportunity to perform experiments in real time and with immediate data transfer to researchers on the ground. Collaboration and interactivity are key to quickly understand the issues and tailor the experiments to the specific situation, which will not only provide new scientific insights but will allow advancing of microgravity research.

## Methods

### Crystallization

Pembrolizumab (human mAb drug substance): the human recombinant IgG4 antibody pembrolizumab was expressed and purified as previously described.^18^ Preparation of PEG 3350 batch crystallization formulations (1 ml bottles): to a 1.5 ml Eppendorf tube were added 333 μl of a solution of pembrolizumab at 20–40 mg/ml, in 20 mM histidine, pH 5.4, followed by the addition of 666 μl of 10.18% PEG 3350 in 50 mM HEPES, pH 7.7, and 100 μl of 2.5% caffeine in 20 mM histidine, pH 5.4. The mixture was vortexed and added to 1 ml PCF bottle at room temperature.

### SONICC imaging

SONICC is an imaging technology for finding, visualizing, and identifying protein crystals. Two technologies, SHG and UV-TPEF, are combined to positively identify protein crystals. All the HH-PCF bottle suspensions from flight and ground were imaged at visible 5 MP images at ×200 magnification, UV-TPEF standard setting 110 mW, and SHG high-power setting 450 mW. Crystallinity was verified by Formulatrix SONICC™ analyses on samples of each bottle analyzed in a Whatman Fast Frame 4 slide well plate after a 1:10 dilution with 10% PEG 3350, 50 mM HEPES, pH 7.0. Crystals appear white against a black background, enabling the identification of crystals even in murky environments. SONICC can detect extremely thin crystals (microcrystals <1 μm).

### Particle size analyses

The Horiba LA-960 uses a laser diffraction particle size-distribution analyses technique to measure suspension particles ranging from 10 nm to 5 mm. The data are presented graphically as *q*%; the density distribution at a diameter size; and the diameters are represented as D50. Representative HH-PCF bottle suspensions from each stack of flight and ground were analyzed in triplicate. Sample preparation: 10 ml of 50 mM HEPES, pH 7.0, 10% PEG 3350, buffer was placed in a 10 ml stirred cuvette and measured for a background measurement on the Horiba LA-960. Ten microliters of concentrated 50 mg/ml samples was dispersed into 10 ml of the 50 mM HEPES, pH 7.0, 10% PEG 3350 buffer, and mixed thoroughly to yield an even suspension.

### Sample preparation for biophysical studies

The content from each bottle was aspirated and dispensed 7–8 times using a 1 ml pipette to insure transfer of the entire bottle content to a 2 ml sterile sample tube. Tubes were centrifuged in a microfuge (Eppendorf) at 3000 r.p.m. for 10 min. The supernatant was removed by aspiration. The pellets were re-suspended in the same PEG formulation (10% PEG 3350, 50 mM HEPES, pH 7.0) and re-centrifuged. This wash procedure was repeated two times. The resulting final pellets were dissolved in 1 ml of 20 mM histidine buffer, pH 5.4, and labeled dissolved crystals (DCs). The DC samples were dialyzed in 2 ml dialysis devices (Float-A-Lyzer) MECO 8–10KD part # G235031 vs. 1 L of 20 mM histidine buffer, pH 5.4 @ 4 °C for 18 h and then re-dialyzed for additional 18 h with fresh dialysate.

### Protein determination (HH-PCF experiments)

Direct A280-based measurement using a NanoDrop UV spectrophotometer. Using the protein content determined by amino acid analysis, a molar absorptivity (extinction coefficient) of 209,155 ± 879 M^−1^∗cm^−1^ at 278 nm was determined, which corresponds to an absorptivity of 1.43 ± 0.006 l/(g∗cm). This value was used for standard pembrolizumab samples. Whole contents from a representative bottle from each stack of identical experiments were centrifuged using low-speed centrifugation. The resulting supernatant was removed by aspiration. The resulting pellet was dissolved in 20 mM histidine buffer, pH 5.4, and then dialyzed vs. 20 mM histidine buffer pH 5.4 (three times). The resulting dialysate was centrifuged to remove particulates.

### Bioassay (HH-PCF experiments)

The pembrolizumab competitive binding ELISA evaluates the ability of pembrolizumab to compete with PD-L1 ligand for binding to PD-1/Fc immobilized on an ELISA plate. The pembrolizumab reference material and test samples (space samples) were serially diluted and mixed with an equal volume of rhB7-H1/Fc chimera (PDL-1) dilution before transfer to ELISA plates. The levels of PD-L1 bound to PD-1/Fc were detected by biotinylated anti-PDL-1, following conjugation with streptavidin and chemiluminescence substrate. Luminescence was measured using a microplate reader and the resulting inhibition response curves were analyzed with the curve fitting software (e.g., SoftMax Pro). The IC50 (half-maximal inhibitory concentration) values generated from this assay are a measurement of the ability of pembrolizumab to inhibit PD-L1 binding to PD-1/Fc. Biological potency is expressed as % relative potency of pembrolizumab reference material. Geometric mean of relative potency from multiple replicates (*N* = 3) of the same sample is reported in Fig. 5 with %GSD and 95% confidence interval.

### Dynamic viscosity measurements

The crystalline contents of five bottles from both the ground and flight experiments were combined and concentrated 5-fold by centrifugation using a Beckman Coulter Allegra X-15R swinging bucket centrifuge and SX4750A rotor at 2095 relative centrifugal force for 5 min and then removing ~4 ml of supernatant. The protein concentration of the resulting concentrated suspensions from both the flight and ground experiments were measured based on dissolution of a 10 μl aliquot in 1 ml normal saline phosphate buffer (ground; 140 mg/ml, 0.5 ml sample; flight; 138 mg/ml, 0.5 ml sample). Samples for dynamic viscosity measurements were prepared by dilution of the stock solution to 50 and 75 mg/ml using 20 mM HEPES buffer, pH 6.8, 10% PEG 3350 for both the concentrated ground and flight samples. A Rheosense m-VROC^TM^ instrument was utilized derives viscosity from the pressure drop using the Hagen–Poiseuille equation.^25^ Shear sweeps were performed from 1500 to 95,000 (1/s) to measure the dynamic viscosity. The viscosity of 50 and 75 mg/ml crystalline pembrolizumab suspensions from both flight and ground samples were measured and plotted vs. different shear rates using a BD Hypak 1 ml pre-filled syringe with a 27 gauge regular wall (RW) with a 1/2 in. needle. Viscosity vs. shear rate data was measured; ground 50 mg/ml 5.48 ± 0.23 vs. flight 50 mg/ml 3.66 ± 0.20 cP and the 75 mg/ml samples 6.83 ± 0.10 ground vs. flight 4.80 ± 0.01 cP (Table 1).

### Dynamic light scattering measurements

One hundred microliters of 50 mg/ml ground and flight concentrated samples were suspended in 0.9 ml of normal saline phosphate buffer. The samples dissolved within 5 min at room temperature. The samples were filtered using a 0.2 μm spin filter (5 K r.p.m.) for 5 min in an Eppendorf MiniSpin microfuge at room temperature. Ten microliters of samples in a cuvette were analyzed using a Wyatt Dynapro Nanostar Dynamic Light Scattering Instrument by taking an average of 10 measurements and visualizing the results using the regulation graph function. The average MW and % polydispersity were recorded for all measurements.

### Sedimentation time analyses

For each sample, a 10 ml graduated cylinder filled to 10 ml of PEG stabilizing buffer was used: a 20 µl sample of the 50 mg/ml crystalline suspensions was layered just below the meniscus and the sedimentation time was measured by visual observation.

### Experiment execution

Each HH-PCF assembly (flight and ground) required a full day for sample filling and hardware integration on 13–14 February 2017. All hardware was filled at the Space Life Sciences Lab (SLSL). The hardware was integrated and transferred to a +4 °C incubator. The flight hardware was turned over to Cold Stowage on 16 February 2017 and loaded into the Polar incubator on the same day. The ground control hardware was maintained in a +4 °C incubator at the SLSL. The MSD PCG (CASIS PCG-5) payload was launched on 19 February 2017 on SpaceX-10 and was transferred to the ISS-NL on 21 February 2017. It was transferred to a MERLIN at +4 °C for 24 h before starting the temperature ramp up. The MERLIN temperature reached +30 °C at 12:30 p.m. EST on 27 February 2017. Ground control hardware remained in an SLSL incubator at +4 °C and the same incubator was used for ground control temperature ramp up on the same schedule used for the MERLIN. On 17 March 2017, the Flight HD-PCG removal from the MERLIN and transfer to a Cargo Transfer Bag was completed at 3:22 p.m. EST. The Ground control hardware transferred to ambient at 3:28 p.m. EST. Following SpaceX-10 splashdown in the Pacific Ocean on 19 March 2017, the MSD PCG-5 hardware was transferred to the Long Beach Airport on 20th March. The hardware was received by MSD from NASA. The flight hardware was hand-carried from Los Angeles to Orlando on 21st March. The ground control hardware was transferred at the Orlando airport and continued to New Jersey, arriving in the evening of 21st March. There were no pre-flight, in-flight, or post-flight anomalies.

### Reporting summary

Further information on research design is available in the Nature Research Reporting Summary linked to this article.

## Supplementary information

Reporting Summary Checklist

## Data Availability

The data that supports the findings of this study are available from the corresponding author upon request. Additional information concerning the materials used for this study will be provided by the authors upon request.
